# Supplementary material for: Atoh8 acts as a regulator of chondrocyte proliferation and differentiation in endochondral bones
Source: PLoS One. 2019 Aug 26;14(8):e0218230. doi: 10.1371/journal.pone.0218230 (PMC6709907; doi:10.1371/journal.pone.0218230)
Supplement: S1 Fig — Schematic representation of wild-type (A), targeted (B) and recombined (C) Atoh8 gene locus. Exon 1 is flanked by two loxP sites [17]. Genotyping primers are shown as black arrows, the length of corresponding PCR products in bp are listed in between. (PDF) [file pone.0218230.s001.pdf]

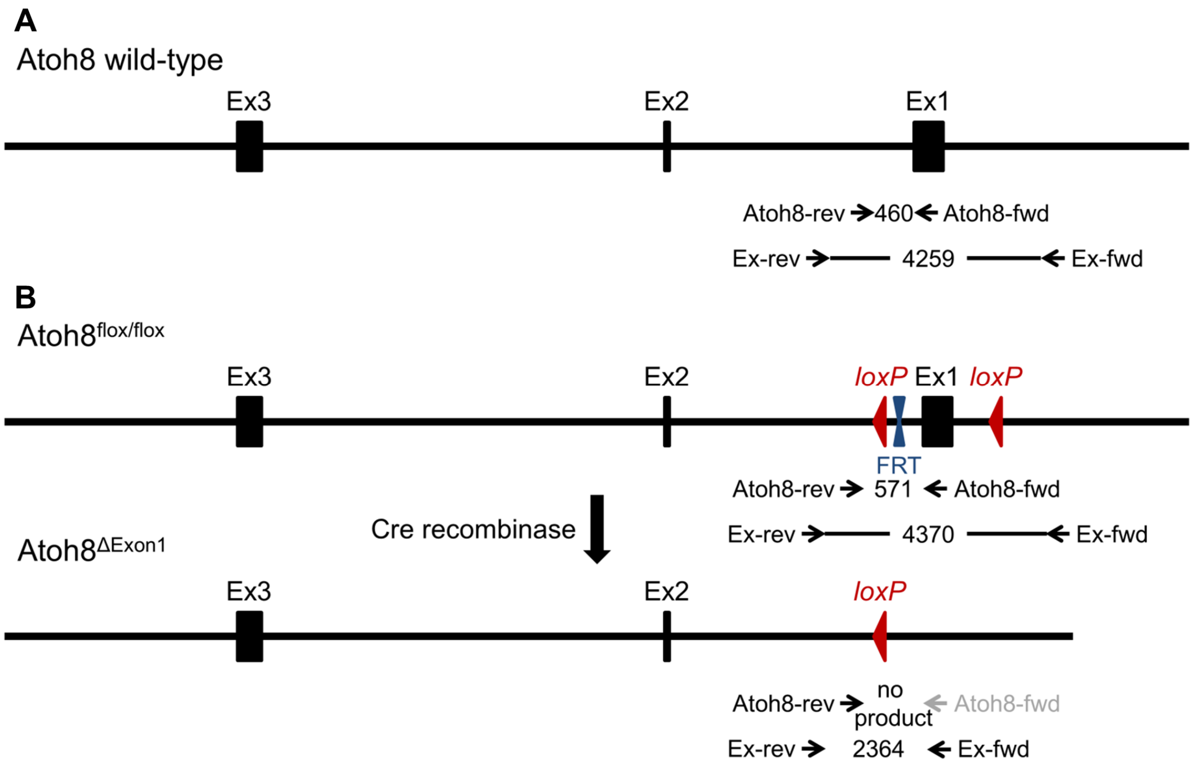

Fig S1: Targeting strategy for Atoh8 deletion in mice. Schematic representation of wild-type (A), targeted (B) and recombined (C) *Atoh8* gene locus. Exon 1 is flanked by two *loxP* sites [17]. Genotyping primers are shown as black arrows, the length of corresponding PCR products in bp are listed in between.
