# Supplementary material for: Atoh8 acts as a regulator of chondrocyte proliferation and differentiation in endochondral bones
Source: PLoS One. 2019 Aug 26;14(8):e0218230. doi: 10.1371/journal.pone.0218230 (PMC6709907; doi:10.1371/journal.pone.0218230)
Supplement: S2 Fig — (A) Radius length of P7 control, Atoh8flox/+;Col2a1-Cre and Atoh8flox/flox;Col2a1-Cre mice. (B, C) Radius length of E14.5 (B) and E16.5 (C) control, Atoh8flox/+;Prx1-Crefemale and Atoh8flox/flox;Prx1-Crefemale mice. (A) n = 10 control, 6 Atoh8flox/+;Col2a1-Cre and 8 Atoh8flox/flox;Col2a1-Cre mice from 5 litters; (B) n = 18 control, 8 Atoh8flox/+;Col2a1-Cre and 17 Atoh8flox/flox;Prx1-Crefemale mice from 8 litters; (C) n = 5 control, 3 Atoh8flox/+;Col2a1-Cre and 5 Atoh8flox/flox;Prx1-Crefemale mice from 2 litters; Bayesian analysis. (PDF) [file pone.0218230.s002.pdf]

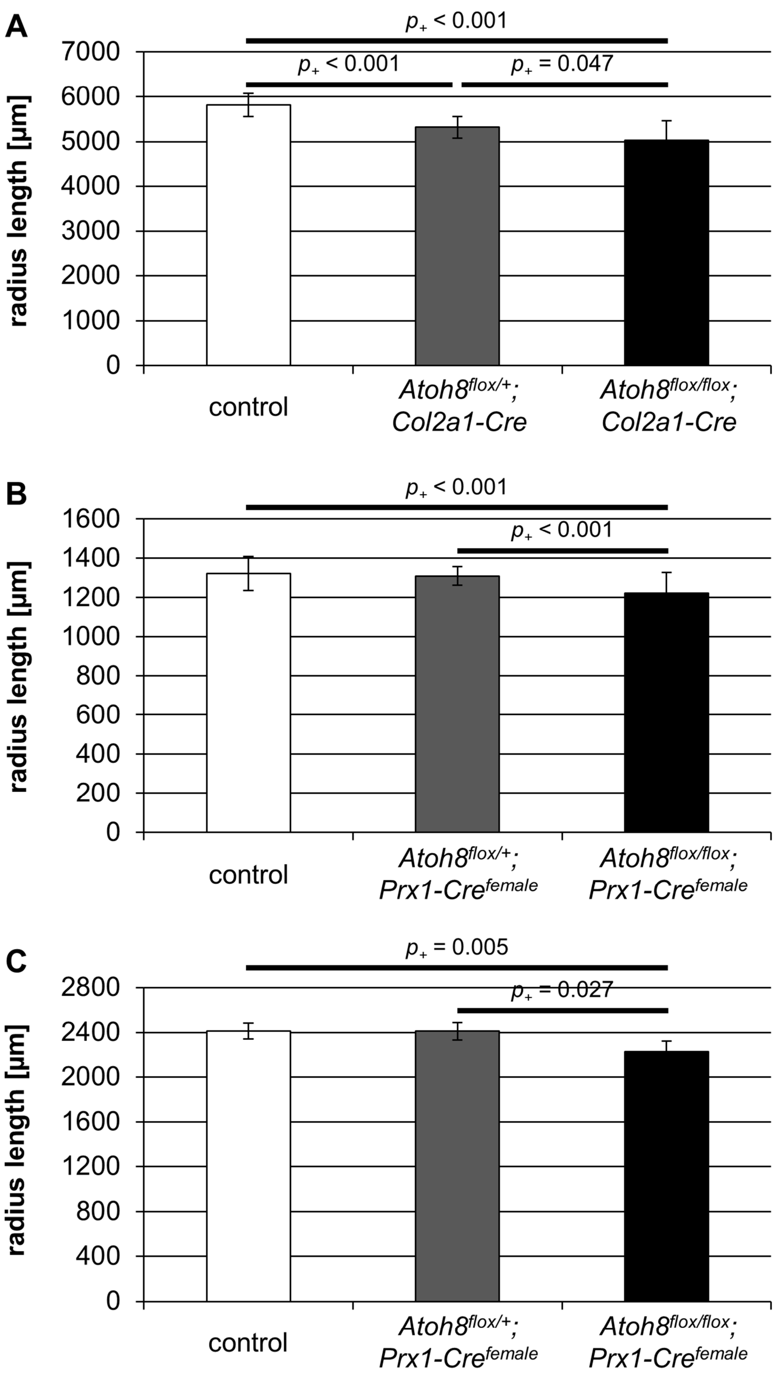

S2 Fig. Comparison of control, heterozygous and homozygous Atoh8 deleted mice. (A) Radius length of P7 control, *Atoh8$^{flox/+}$;Col2a1-Cre* and *Atoh8$^{flox/flox}$;Col2a1-Cre* mice. (B, C) Radius length of E14.5 (B) and E16.5 (C) control, *Atoh8$^{flox/+}$;Prx1-Cre^{female}* and *Atoh8$^{flox/flox}$;Prx1-Cre^{female}* mice. (A) n = 10 control, 6 *Atoh8$^{flox/+}$;Col2a1-Cre* and 8 *Atoh8$^{flox/flox}$;Col2a1-Cre* mice from 5 litters; (B) n = 18 control, 8 *Atoh8$^{flox/+}$;Col2a1-Cre* and 17 *Atoh8$^{flox/flox}$;Prx1-Cre^{female}* mice from 8 litters; (C) n = 5 control, 3 *Atoh8$^{flox/+}$;Col2a1-Cre* and 5 *Atoh8$^{flox/flox}$;Prx1-Cre^{female}* mice from 2 litters; Bayesian analysis.
