# Supplementary material for: Atoh8 acts as a regulator of chondrocyte proliferation and differentiation in endochondral bones
Source: PLoS One. 2019 Aug 26;14(8):e0218230. doi: 10.1371/journal.pone.0218230 (PMC6709907; doi:10.1371/journal.pone.0218230)
Supplement: S3 Fig — Radius length of E16.5 control and Atoh8flox/flox;Col2a1-Cre mice are comparable. n = 2 control and Atoh8flox/flox;Col2a1-Cre mice from 2 litters; Bayesian analysis. (PDF) [file pone.0218230.s003.pdf]

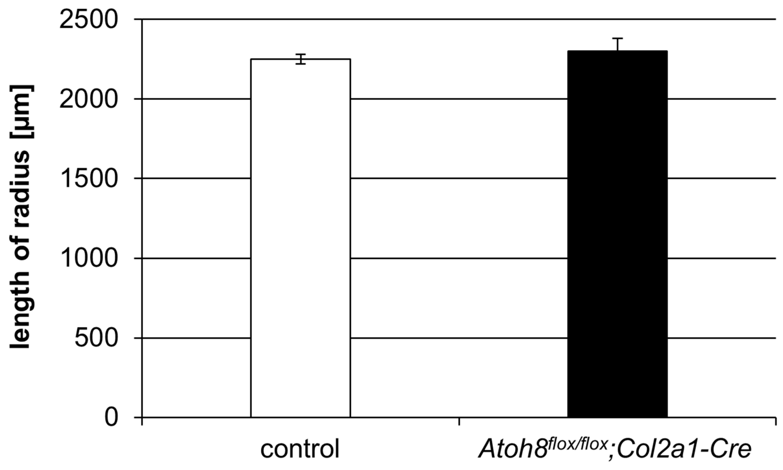

S3 Fig. Radius length of prenatal *Atoh8*^{flox,flox}*;Col2a1-Cre* mice is not reduced. Radius length of E16.5 control and *Atoh8*$^{flox/flox}$*;Col2a1-Cre* mice are comparable. n = 2 control and *Atoh8*$^{flox/flox}$*;Col2a1-Cre* mice from 2 litters; Bayesian analysis.
