# Supplementary material for: Atoh8 acts as a regulator of chondrocyte proliferation and differentiation in endochondral bones
Source: PLoS One. 2019 Aug 26;14(8):e0218230. doi: 10.1371/journal.pone.0218230 (PMC6709907; doi:10.1371/journal.pone.0218230)
Supplement: S4 Fig — Relative Atoh1 (A), Atoh2 (B), Atoh3 (C), Atoh4 (D), Atoh5 (E), Atoh7 (F) and Atoh8 (G) mRNA expression of NMRI embryos (grey) or control (white) and Atoh8flox/+;Prx1-Crefemale (black) forelimb skeletal elements. n = 2 wild-type mice from 2 litters; n = 4 control and 3 Atoh8flox/+;Prx1-Crefemale mice from 2 litters. (PDF) [file pone.0218230.s004.pdf]

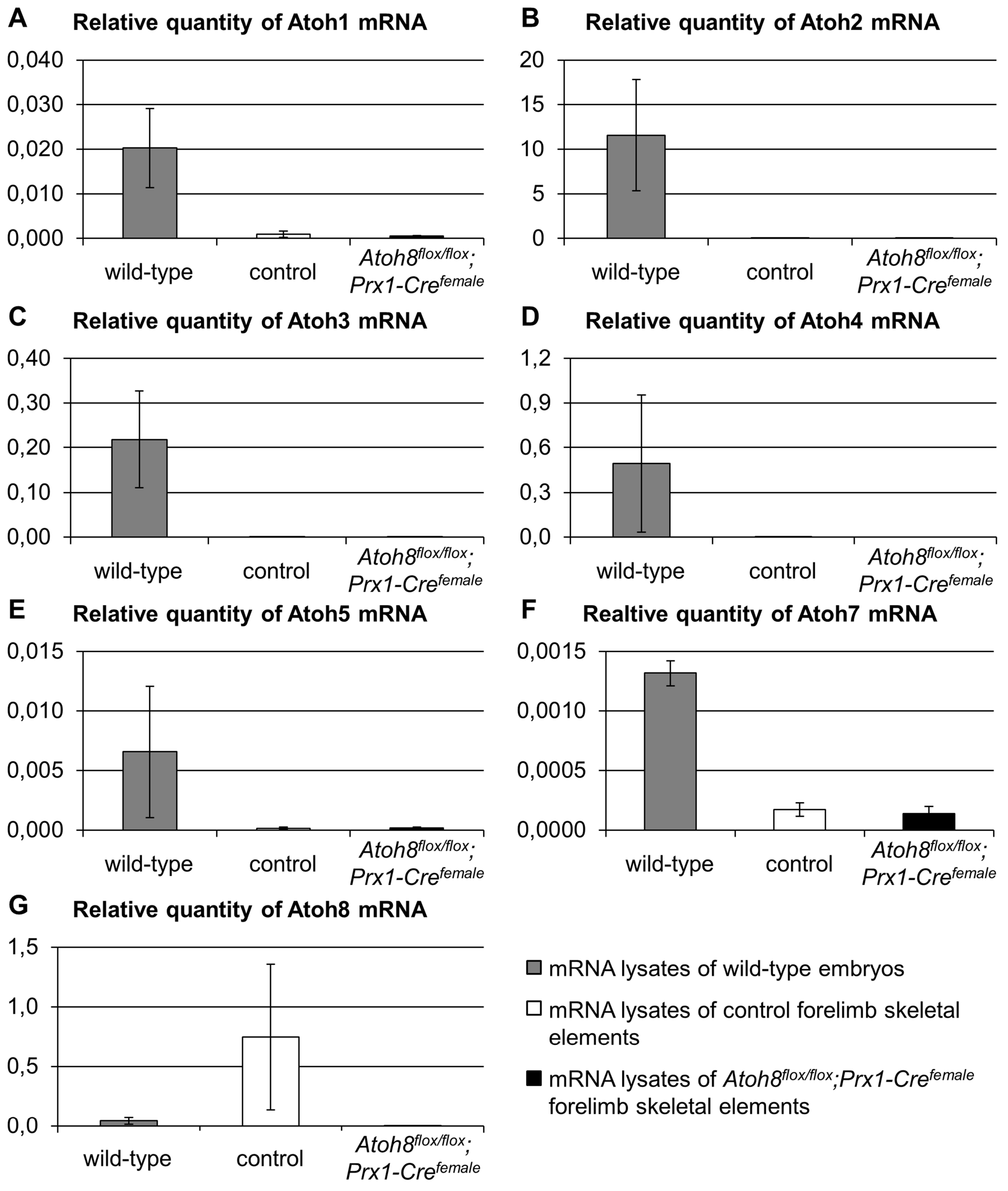

S4 Fig. *Atoh8* is the only *Atoh* gene which is noteworthy expressed in chondrocytes. Relative Atoh1 (A), Atoh2 (B), Atoh3 (C), Atoh4 (D), Atoh5 (E), Atoh7 (F) and Atoh8 (G) mRNA expression of NMRI embryos (grey) or control (white) and *Atoh8*$^{flox/+}$*;Prx1-Cre*^{female} (black) forelimb skeletal elements. n = 2 wild-type mice from 2 litters; n = 4 control and 3 *Atoh8*$^{flox/+}$*;Prx1-Cre*^{female} mice from 2 litters.
