# Supplementary material for: Atoh8 acts as a regulator of chondrocyte proliferation and differentiation in endochondral bones
Source: PLoS One. 2019 Aug 26;14(8):e0218230. doi: 10.1371/journal.pone.0218230 (PMC6709907; doi:10.1371/journal.pone.0218230)
Supplement: S1 Table — The probability of a negative effect (p-) and a positive effect (p+) caused by an Atoh8 deletion or a Purmorphamine treatment were determined by Bayesian analysis. All calculated p- and p+ values for the different experiments are listed in this table. (PDF) [file pone.0218230.s005.pdf]

Tab. S1: Overview about calculated p_- and p_+ values. The probability of a negative effect (p_-) and a positive effect (p_+) caused by an Atoh8 deletion or a Purmorphamine treatment were determined by Bayesian analysis. All calculated p_- and p_+ values for the different experiments are listed in this table.

**Measurement**	**Fig.**	p_-	p_+
Relative quantity of Col2 mRNA in micromass cultures day 7 (*Atoh8*$^{flox/flox}$*;Prx1-Cre*^{female} vs. control)	1G	0.984	0.016
Relative quantity of Col2 mRNA in micromass cultures day 14 (*Atoh8*$^{flox/flox}$*;Prx1-Cre*^{female} vs. control)	1G	0.994	0.006
Relative quantity of Col2 mRNA in micromass cultures day 21 (*Atoh8*$^{flox/flox}$*;Prx1-Cre*^{female} vs. control)	1G	0.880	0.120
Relative quantity of Col2 mRNA in micromass cultures day 28 (*Atoh8*$^{flox/flox}$*;Prx1-Cre*^{female} vs. control)	1G	0.797	0.203
Radius length P7 (*Atoh8*$^{flox/flox}$*;Col2a1-Cre* vs. control)	2C	> 0.999	< 0.001
Radius length P14 (*Atoh8*$^{flox/flox}$*;Col2a1-Cre* vs. control)	2C	> 0.999	< 0.001
Radius length 6 month (*Atoh8*$^{flox/flox}$*;Col2a1-Cre* vs. control)	2C	0.997	0.003
Length Col2 P7 (*Atoh8*$^{flox/flox}$*;Col2a1-Cre* vs. control)	3D	0.991	0.009
Distance Col10 P7 (*Atoh8*$^{flox/flox}$*;Col2a1-Cre* vs. control)	3D	0.975	0.025
Length Col10 P7 (*Atoh8*$^{flox/flox}$*;Col2a1-Cre* vs. control)	3D	0.978	0.022
Proliferation of round chondrocytes P7 (*Atoh8*$^{flox/flox}$*;Col2a1-Cre* vs. control)	3F	0.997	0.003
Proliferation of columnar chondrocytes P7 (*Atoh8*$^{flox/flox}$*;Col2a1-Cre* vs. control)	3F	0.996	0.004
Radius length E16.5 (*Atoh8*$^{flox/flox}$*;Prx1-Cre*^{female} vs. control)	4B	0.995	0.005
Length Col2 E16.5 (*Atoh8*$^{flox/flox}$*;Prx1-Cre*^{female} vs. control)	4E	0.984	0.016
Distance Ihh E16.5 (*Atoh8*$^{flox/flox}$*;Prx1-Cre*^{female} vs. control)	4E	0.980	0.020
Length Ihh E16.5 (*Atoh8*$^{flox/flox}$*;Prx1-Cre*^{female} vs. control)	4E	0.947	0.053
Distance Col10 E16.5 (*Atoh8*$^{flox/flox}$*;Prx1-Cre*^{female} vs. control)	4E	> 0.999	< 0.001
Length Col10 E16.5 (*Atoh8*$^{flox/flox}$*;Prx1-Cre*^{female} vs. control)	4E	0.938	0.062
Radius length E14.5 (*Atoh8*$^{flox/flox}$*;Prx1-Cre*^{female} vs. control)	5B	> 0.999	< 0.001
Length zone of PC E14.5 (*Atoh8*$^{flox/flox}$*;Prx1-Cre*^{female} vs. control)	5B	0.994	0.006
length zone of HC E14.5 (*Atoh8*$^{flox/flox}$*;Prx1-Cre*^{female} vs. control)	5B	> 0.999	< 0.001
Proliferation of round chondrocytes E14.5 (*Atoh8*$^{flox/flox}$*;Prx1-Cre*^{female} vs. control)	5D	0.256	0.744
Proliferation of columnar chondrocytes E14.5 (*Atoh8*$^{flox/flox}$*;Prx1-Cre*^{female} vs. control)	5D	> 0.999	< 0.001
Length Col2 control limb cultures (Purmorphamine vs. DMSO)	7B	< 0.001	> 0.999
Length Col2 *Atoh8*$^{flox/flox}$*;Prx1-Cre*^{female} limb cultures (Purmorphamine vs. DMSO)	7B	< 0.001	> 0.999

**Measurement**	**Fig.**	p_-	p_+
Effect of Purmorhamine treatment on length Col2 (*Atoh8*$^{flox/flox}$*;Prx1-Cre*^{female} vs. control)	7B	0.320	0.680
Proliferation of round chondrocytes control limb cultures (Purmorphamine vs. DMSO)	7D	< 0.001	> 0.999
Proliferation of columnar chondrocytes control limb cultures (Purmorphamine vs. DMSO)	7D	< 0.001	> 0.999
Proliferation of round chondrocytes *Atoh8*$^{flox/flox}$*;Prx1-Cre*^{female} limb cultures (Purmorphamine vs. DMSO)	7D	0.397	0.603
Proliferation of columnar chondrocytes *Atoh8*$^{flox/flox}$*;Prx1-Cre*^{female} limb cultures (Purmorphamine vs. DMSO)	7D	0.010	0.99 0
Effect of Purmorhamine treatment on proliferation of round chondrocytes (*Atoh8*$^{flox/flox}$*;Prx1-Cre*^{female} vs. control)	7D	> 0.999	< 0.001
Effect of Purmorhamine treatment on proliferation of columnar chondrocytes (*Atoh8*$^{flox/flox}$*;Prx1-Cre*^{female} vs. control)	7D	> 0.999	< 0.001
Relative quantity of Ihh mRNA E14.5 (*Atoh8*$^{flox/flox}$*;Prx1-Cre*^{female} vs. control)	7F	0.986	0.014
Relative quantity of Ihh mRNA E16.5 (*Atoh8*$^{flox/flox}$*;Prx1-Cre*^{female} vs. control)	7F	0.992	0.008
Radius length P7 (*Atoh8*$^{flox/flox}$*;Col2a1-Cre* vs. control)	S2A	> 0.999	< 0.001
Radius length P7 (*Atoh8*$^{flox/+}$*;Col2a1-Cre* vs. control)	S2A	> 0.999	< 0.001
Radius length P7 (*Atoh8*$^{flox/flox}$*;Col2a1-Cre* vs. *Atoh8*$^{flox/+}$*;Col2a1-Cre*)	S2A	0.953	0.047
Radius length E14.5 (*Atoh8*$^{flox/flox}$*;Prx1-Cre*^{female} vs. control)	S2B	> 0.999	< 0.001
Radius length E14.5 (*Atoh8*$^{flox/+}$*;Prx1-Cre*^{female} vs. control)	S2B	0.070	0.930
Radius length E14.5 (*Atoh8*$^{flox/flox}$*;Prx1-Cre*^{female} vs. *Atoh8*$^{flox/+}$*;Prx1-Cre*^{female})	S2B	> 0.999	< 0.001
Radius length E16.5 (*Atoh8*$^{flox/flox}$*;Prx1-Cre*^{female} vs. control)	S2C	0.995	0.005
Radius length E16.5 (*Atoh8*$^{flox/+}$*;Prx1-Cre*^{female} vs. control)	S2C	0.600	0.400
Radius length E16.5 (*Atoh8*$^{flox/flox}$*;Prx1-Cre*^{female} vs. *Atoh8*$^{flox/+}$*;Prx1-Cre*^{female})	S2C	0.973	0.027
Radius length E16.5 (*Atoh8*$^{flox/flox}$*;Col2a1-Cre* vs. control)	S3	0.393	0.607
