# Supplementary material for: Atoh8 acts as a regulator of chondrocyte proliferation and differentiation in endochondral bones
Source: PLoS One. 2019 Aug 26;14(8):e0218230. doi: 10.1371/journal.pone.0218230 (PMC6709907; doi:10.1371/journal.pone.0218230)
Supplement: S2 Table — (PDF) [file pone.0218230.s006.pdf]

S2 Table. Overview of additional primer pairs used for the expression analysis of the different *Atoh* genes by qRT-PCR.

**Primer**	**Sequenz (5` – 3`)**
Atoh1 forward	CCC TAA CAG CGA TGA TGG CA
Atoh1 reverse	AGG GAT ATT TGT CAC GGG GC
Atoh2 forward	ATG CGA CAC TCA GCC TGA AA
Atoh2 reverse	CTG GGA TTC GGG CAT TAC GA
Atoh3 forward	TGC CCA GAG ACT GTG GTA CT
Atoh3 reverse	AAG AGC CCG GTC TTC TCT CT
Atoh4 forward	GCA CGA GAA CGA CAA CAC AC
Atoh4 reverse	TTG ACG AAC ATC CTA CGC GG
Atoh5 forward	ACG CAA TTT ACT CCA GGC GA
Atoh5 reverse	GAG GCG CCA TCC TAG TTC TC
Atoh7 forward	CTA CAT CAT CGC GCT CAC CC
Atoh7 reverse	TCA TGA AAC CTG GAG CCT AGC
